# Myths and Fallacies about Male Contraceptive Methods: A Qualitative Study amongst Married Youth in Slums of Karachi, Pakistan

**DOI:** 10.5539/gjhs.v5n2p84

**Published:** 2012-12-04

**Authors:** Noureen Nishtar, Neelofar Sami, Anum Faruqi, Shaneela Khowaja, Farid-Ul-Hasnain

**Affiliations:** 1 Health Systems Division, Aga Khan University, Karachi, Pakistan; 2 Willows Foundation, Pakistan; 3 Family Medicine Department, Aga Khan University, Karachi, Pakistan; 4 School of Nursing, Aga Khan University, Karachi, Pakistan; 5 Reproductive Health Division, Aga Khan University, Karachi, Pakistan

**Keywords:** male contraceptives, youth, Pakistan, myths, fallacies

## Abstract

Pakistan presently has one of the largest cohorts of young people in its history, with around 36 million people between the ages of 15 and 24 years. One of the main reasons for high population growth in Pakistan is almost stagnant contraceptive prevalence rate of 30% nationally and 17.4% amongst youth. The study was conducted to explore the perceptions regarding myths and fallacies related to male contraceptive methods among married youth aged 18-24 year in Karachi, Pakistan. Qualitative exploratory study design was adopted and a total of eight Focus Group Discussions (FGDs) were conducted. Study was conducted in two Union Councils of Korangi Town in the squatter settlement of Karachi, Pakistan from July to September 2010. Thematic analysis was done manually. General, physical, sexual, psychological, socio-cultural and religious were the common categories which lead to myths and fallacies related to condoms use and vasectomy among the married youth. The foremost myth amongst male and female youth was that use of both condoms and vasectomy cause impotence in males. Additionally, condoms were thought to cause infections, backache and headache in males. Some youth of the area think that vasectomy is meant for prisoners only. In conclusion our findings suggest that the potential reasons behind low use of male contraceptive methods among youth of squatter settlement of Karachi were myths and fallacies about male contraceptive methods. There are some important policy implications like counseling of the couple through peers and well trained family planning service providers to address these myths and misconceptions from the minds of youth.

## 1. Introduction

In Pakistan, the concept of family planning is rooted in and is surrounded by its traditional value system in which male members of society and mothers-in-law are mostly the main decision makers (Agha, 2010) and childbearing women have barely any control in adopting the contraception (Saleem & Isa, 2004). Whereas on policy and collective action plans the government and administrative machinery lacks political will to address the issue as the government policies do not see any priorities to address them (Khan, 1999). Therefore, despite rigorous efforts and strategies initiated by various agencies fewer positive results have emerged in improving contraceptive prevalence rate (CPR) in Pakistan (Cleland et al., 2006; Sirageldin, Norris, & Hardee, 1976). In the same context some of the population and health indicators are shown in table 1 highlighting some immediate short and long term policy interventions to improve CPR specifically amongst youth.

**Table 1:** Pakistan health indicators

Total population	185 Millions*
Population growth rate	1.9**
Total fertility rate (TFR)	4.1**
Population of youth	36 Millions*
Maternal Mortality Ratio (MMR)	297/10,000 live births**
National Contraceptive Prevalence Rate	30*
Contraceptive Prevalence Rate among youth	17.4***

Sources:**: Pakistan Demographic and Health Survey 2006-7: National Institute of Population Studies, Islamabad, Pakistan.

* World Population Report 2010. Population Reference Bureau. Washington DC. 2010.

*** UNFPA Survey on adolescents and youth 2006.

According to 2007 data, Pakistan's fertility rate is at 3.3 children in urban settings and 4.5 children in rural areas and contraception use is around 30% (Pakistan Demographic Health Survey 2006-7). And population growth rate is 1.9% (Pakistan Demographic Health Survey 2006-7). Various national, regional and international empirical evidences have recommended numerous interventions and strategies in catering the issue of birth spacing and one of its major recommendations is the utilization of family planning methods by male partner (Smith et al 2009, Terefe & Larson, 1993). Moreover, family planning saves lives and promotes reproductive health by decreasing the prevalence of HIV/AIDS and other sexually transmitted infections (DiClemente, 2004; Smith et al., 2009). Thus, if males will adopt the contraceptive methods, it will contribute in improving the birth outcome by giving benefits not only to the female partner on one hand but will also have positive impact on the health of family on the other.

Whereby the term ‘fallacy’ is mostly used to indicate any false belief and is an error in reasoning (Quereshi & Sheikh, 2006), myths are often collectively shared fantasies that contribute to the emotional strength of both individuals and the society (Neises, 2000). People in every culture have an accumulation of myths and fallacies, which could be social, cultural or biological (Quereshi & Sheikh, 2006). This collective ignorance and lack of understandings create obstacles in achieving the targets set in Millennium Development Goals (MDGs) 4, 5 & 6. There are also various myths and fallacies associated with use of condoms (IPPF, 2007). These include pain, bleeding, infertility, infection, cancer, back or kidney pain and even death in men with condom's use (Jackson 2004). The fact is that condom does not cause any of these complications (Jackson, 2004). There could be other justifications for some of the side effects. For instance, excessive rubbing of the penis against a dry vagina can be painful for the woman and sometimes for the man as well, and could lead to vaginal bleeding too (Jackson, 2004). Some of the fallacies about vasectomy are that men lose their sexual powers and become impotent after this procedure. Some people also think that after vasectomy, the marital life of couple is not pleasurable (Aguilar, 2005). The fact is that after vasectomy, sperm continues to accumulate in the testicles as usual and semen continues to be secreted. The only difference is that the tubes no longer conduct the sperm to the semen (IPPF, 2007). Thus, male sperm cannot meet with the egg; other than that the sexual relations remain normal. It is also believed that men lose their physical strength due to vasectomy (Aguilar, 2005). In reality evidence based studies have indicated that there is no relationship between vasectomy and loss of working ability. Any decrease in ability to work could be an emotional effect (IPPF, 2007).

36 million of population in Pakistan is youth (PRB, 2010). And it has been seen that use of family planning methods amongst youth are much lower at 17.4% (UNFPA, 2006-7) than the national average of 30% (PDHS, 2006). Nonetheless, being a patriarchal and culturally rooted society, male-participation is not considered as a positive and acceptable practice in using family planning methods (Azmat, 2011). Moreover, due to these cultural barriers most of our health care system is emphasizing on the strategies for the female family planning methods and prevalence of condom use is only 6.8 percent in Pakistan (PDHS, 2006). Low CPR could be due to myths and fallacies about male contraceptive methods (IPPF, 2007). These myths and fallacies could be due to certain socio-cultural reasons and exaggerated side effects of these contraceptive methods (Casterline, 2001; Mahmood & Ringheim, 1996; Stephenson & Hennick, 2004). Therefore, there is an immense need to understand the myths and fallacies about male family planning methods use amongst youth. Moreover it is generally assumed that youth will not opt for vasectomy due to their age. But it is important to remove the myths associated with the method so that when they enter into an age where they can opt for the method, they don’t have any false beliefs. To researcher's knowledge, no study has been done which emphasis on these aspects on youth in Pakistan. The current study is geared towards understanding males’ attitudes and beliefs related to family planning methods and draw policy implications related to the topic. The study therefore explored the perceptions regarding myths and fallacies about male family planning methods amongst currently married youth aged 18-24 years of Karachi

## 2. Methodology

### 2.1 Study Design

An exploratory qualitative study was conducted to achieve the aforementioned objectives. As Family planning is a sensitive issue in Pakistani context due to social, cultural and religious concerns (Ali et al., 2004; Kadir et al., 2003; Nasir & Hinde, 2011) and in this study, we wanted to catch the participants viewpoints, perceptions and ideas on myths and fallacies about male contraceptive methods. FGDs are more geared to explore the sensitive issues; therefore FGDs were conducted with youth. As this is a sensitive topic, young people in Pakistan would be reluctant to discuss such matters in individual interviews (Dahlgern, Emmilin & Winkvist, 2004). In this regard, 8 sex selective focus group discussions (FGDs), 4 each with male and female participants up till point of saturation were conducted. Due to cultural sensitivity male moderator and note-taker conducted the male FGDs and female moderator and note taker conducted the female FGDs. Purposive sampling was the strategy used for conducting focus group discussions with the youth.

### 2.2 Study Setting

The study was conducted in Karachi Pakistan between July to October, 2010. Situated in the North-Western part of South Asia, Pakistan, with about 185 million people (PRB, 2010), is bordered by China on the northeast side, India on the eastern side, Iran and Afghanistan on the western side and the Arabian Sea on its South. Karachi, with 16 million people, is the largest city of the country, main port and hub of national industrial, commercial and financial activities. Therefore, people from all groups and ethnicities live in the city. In terms of income, people from all strata live in the city in various high, middle or low income neighborhoods. For study purposes Union Council UC 2 (Nasir Colony) and UC 3 (Chakra Goth) were selected because majority of the residents there belong to low income strata and with relatively lesser education. UC is the primary governmental body at the lowest step of the local government institutions and it has usually a population of about 55,000 to 60,000. Literature has shown that use of contraceptive among couples belonging to lower income strata and having lesser education is comparatively lower with usually more children in general (Saleem & Bobak, 2005). Therefore selection of such group would provide a better insight into the objectives of the study and more explicitly.

### 2.3 Study Participants

The study participants included currently married males and females aged 18-24 years, having at least one live child and residents of 2 UCs of Korangi.

### 2.4 Focus Group Discussions

Lady health workers working in the area facilitated the identification of participants and arranged the male and female focus group discussions at their own houses, which are called “Health Houses”. FGD Guidelines were developed after extensive literature search keeping in mind the study question and objectives along with repeated consultations with experts in the subject. Guidelines for FGDs were pilot tested in Azam Basti, which is similar to the catchment area socio-demographically. The research team comprised of a male and the female moderator (PI of the study). In addition, a separate male and female note takers were hired for the male and female FGDs respectively. For ensuring validity and audit trail, FGDs were transcribed in Urdu and then translated in English on the same day. Moreover, both moderator and note-taker also share their notes at the end of the FGDs and also share the main findings with the participants of the FGDs.

### 2.5 Ethical Review

Permission for data collection was taken from the Regional Coordinator of National Program for Family Planning and Primary Health Care, Korangi Town, Karachi. Approval for conducting the study was sought from the ethical review committee of Aga Khan University Karachi. Verbal and written consent was taken from all study participants.

### 2.6 Study Analysis

Thematic analysis was the strategy used for analysis of interviews. Thematic analyses supports in the exploration for themes that emerge as pertinent to the phenomenon under description. The practice offers the opportunity for identifying themes through careful reading and rereading of the data (Rice & Ezzy, 1999). Whereby, it is a form of pattern recognition within the data, where emerging themes become the categories for analysis (Fereday & Muir-Cochraine, 2006). Thematic analysis was done by coding of the data. Furthermore categories were made and themes were derived.

### 2.7 Trustworthiness

Transcription was done on the day of the FGDs to ensure that there is minimum time lapse so that the data on tapes is transcribed to the paper with the fresh memory. All of the authors were competent in the local language and the cultural meaning of the content (NN, NS, FH, SK & AF). Recordings and notes were also consulted for any verification at the end of the FGDs. FGDs were transcribed into Urdu and then translated to English by the Principal Investigator. Credibility was maintained by selection of context, informants and well-structured FGD guidelines. Conformability was achieved through separate coding by the first and third author, whereby similarities and dissimilarities were discussed. Consensus on codes and categories was thereafter reached. During the analytical process, the first and third author thoroughly discussed the structure of codes, categories and themes. All authors read, discussed and agreed on the final categorisation and theme. Two of the authors (NN and AF) agreed in the way the codes and categories were labeled and categorized, which in later stages were verified by the other two authors (FH & NS). All FGDs were conducted in Urdu, moderated by local researchers well versed with the context, transcribed into English by the principal investigator and analysed within a week. Verification of transcriptions was done by the first author who listened to the audio-tapes twice.

The quotations given in the study are intended to facilitate the reader's evaluation of the creditability of results (Graneheim & Lundman, 2004).

## 3. Results

A total of eight FGDs were conducted with married youth, four each with males and females. The total number of participants of the FGDs were 50 (26 males and 24 females) with an average of 6-7 participants/FGD.

### 3.1 Demographic Profile of Participants

Demographic and descriptive characteristics about study participants are displayed in Table 2. The mean age of males was 24 ± 1.75 years. Most of them were factory workers by profession while others were drivers, tailors, dyers, mechanic and shopkeepers. Nearly half of them were illiterate while others had completed 5-12 years of schooling. Mean age of female participants was 23±1.50 years. All of them were housewives. Majority of them were also illiterate and few reported to complete 5-10 years of schooling.

**Table 2:** Socio-demographic characteristics of FGDs participants

Characteristics	N=50	
Gender	Males	26
	Females	24

Age in years (18-24 years)	Males	Mean= 24 ± 1.75 years
	Females	Mean= 23±1.50 years

Educational attainment		
None	14	
Up to grade IV	16	
Up to grade VIII	6	
Up to grade IX	3	
Up to grade X	4	
Up to grade XI	3	
Up to grade XII	4	

Occupational status	Non-working	5
	House wives	23
	Working	22

Note: All participants are married with at-least one child

A total of 8 FGDs were conducted with married youth, four each with males and females. The total number of participants of the FGDs were 50 (twenty six males and twenty four females) with an average of 6-7 participants/FGD.

Thematic analysis was done manually keeping objective of the study upfront. Under the major theme of myths and fallacies about male contraceptive methods (condoms and vasectomy), five major categories emerged from the FGD's data: general; physical; sexual; health and psychological and socio-cultural and religious factors contributing to myths and fallacies around condoms use and vasectomy.

### 3.2 These Categories Are Presented Below

#### 3.2.1 General Myths and Fallacies

Most of the male youth were of the view that if family Planning is to be practiced by a couple, prime responsibility for contraceptives use is of the female partner. The females’ general view was that although it was not exclusively their responsibility but since men generally avoid using contraceptives the responsibility of doing something to avoid unintended pregnancies fell upon the shoulders of the females. The females also accepted this as their fate

“This is a male's world. Women in our area don’t want to annoy their husbands; they would rather sacrifice their own health”… (Female participant …FGD 1)

They all agreed that condoms are good option because they are safe both for them and their husbands. We asked about the side effects of condoms and 2-3 women together said that they can leak.

Generally there was no knowledge among male youth about the vasectomy and whatever was known was based on the wrong information that people get from the friends, relatives and acquaintances. There were interesting discussions about vasectomy which revealed that the perception about vasectomy was based on misinformation or disinformation.

**Table 3:** Examples of codes, categories and theme from the thematic analysis of FGDs with male and female participants

Theme	Categories	Codes
Myths and Fallacies about condoms amongst youth	Health factors contributing to myths and fallacies	Cause infections in males as well as females Cause headache in husbands Cause joint pain in husbands Cause backache in husbands and wives Cause leucorrhoea in females
	Sexual factors contributing to myths and fallacies	Men become sterile by using it
	Psychological factors contributing to myths and fallacies	Men don’t find relaxation of mind by using them
	Physical factors contributing to myths and fallacies	Can burst leading to unwanted pregnancy Can leak leading to unwanted pregnancy

Myths and fallacies about vasectomy amongst youth	Physical factors contributing to myths and fallacies	Men loses their physical power and become weak
	Cultural factors contributing to myths and fallacies	It is successful if the man reduces his diet In vasectomy a vein is cut from the toe of the leg Those who are caught for the case of rape, police cut their veins and make them impotent in vasectomy
	Sexual factors contributing to myths and fallacies	Men lose their sexual powers Men will become impotent

#### 3.2.2 Physical Factors

As shown in table 2 there was a consensus among male and female participants that condoms use is linked with laziness and weight gain in both male and females.

“The weight of a person increases and he becomes lazy by using condoms.” … (Male participant…FGD 4)

Females also gave similar views saying that they have heard that men lose their physical powers by vasectomy. Another finding that came forth was a new finding that they have also heard that vasectomy is successful, if males reduce their diets after the procedure.

#### 3.2.3 Health and Psychological Factors

There was a general atmosphere among the participants, both males and females, that use of condoms cause many health problems for both male and female partners. The cited problems included infections, headache and joint pains both in males and females.

“I know condoms are responsible for spreading infections and have heard of similar complaints from a friend that condom causes many health problems including headache and joint pain, as well as various infections in males and females.” … (Female participant…FGD 7)

The majority of the female participants said,

“*Use of the condoms depends on the mood of our husbands*” … *(Female participant…FGD 3)*

Most men thought that the use of condom is harmful for men and chemicals used in its manufacturing and packing causes health hazards particularly inflammation in the different parts of the body.

“*Using condoms is wrong, because they are treated with chemicals which are not good for health and are responsible for swelling in various parts of the body including joints”… (Male participant…FGD.2)*

Moreover condoms used are associated with joint pains in both males and females as perceived by majority of males and females participants as shown in box 1.

#### 3.2.4 Sexual Factors

Most of the participants shared that condoms use could cause loss of sexual pleasure among the users. One striking myth that came to the fore about condoms use was that there was a general understanding amongst the male participants that if men use condoms it can make them impotent and such a development would ruin their lives.

“It's male's world and if at anytime we need to remarry, and if we get sterile by using contraceptive methods, what we will do?” (Male participant…FGD 8)

On further probing about the possible side effects of vasectomy, one participant shared her perception:

“Men lose their sexual power and there is the problem of weight gain and laziness. Maybe they will never have a child. This is the world of males and if afterwards he needs a child, he will not be able to do anything.” (Female participant…FGD 6)

Both groups of respondents also gave similar views by saying that they have heard that men lose their physical powers and become weak by vasectomy.

New myths and fallacies about condoms and vasectomy not found in literature:

- Vasectomy is successful, if males reduce their diets after the procedure
- Condoms use is linked with joint pain in both male and females.
- Vasectomy is done on the prisoners convicted of rape charges. According to them, in this procedure the vein of their big toe is cut and resultantly they permanently become impotent and sterile.
- Vasectomy is for females.

#### 3.2.5 Socio-Cultural and Religious Factors

Generally there was dearth of knowledge among male youth about the vasectomy and whatever was known was based on the wrong information that people get from the friends, relatives and acquaintances. There were interesting discussions about vasectomy which revealed that the perception about vasectomy was based on misinformation or disinformation. As shown in box 1, the statements made by two male participants disclosed their wrong views in this regard that

“*Vasectomy is done on the prisoners convicted of rape charges. According to them, in this procedure the vein of their big toe is cut and resultantly they permanently become impotent and sterile.”* *(Male participant….FGD…)*

“Vasectomy is a procedure in which a capsule is placed in the male's sexual organ and it makes them permanently sterile” (Female participant)

As shown in box 1, one participant said that:

“Vasectomy is usually for ladies and it is not for men” (Male participant)

Some of the females shared their perceptions about the use of condoms and vasectomy, by saying that if someone avoids pregnancy then Allah makes them ill in some other way like blood pressure or diabetes etc.

## 4. Discussion

In order to increase the overall contraceptive prevalence rate and massive population growth in Pakistan, more of the focus is needed on condom use among males, because it is the only available reversible male contraceptive method. Moreover condoms are important in the aspect that use of condoms also saves the couple against sexually transmitted infections (Waller, 2002; Sayegh, 2005). Vasectomy is least likely to be used by male youth but there is a need to study the myths and fallacies associated with it, so as to make long term strategies to address these myths in their minds. Therefore we have attempted to find out the myths and fallacies associated with condoms and vasectomy among youth in this study.

In this study multiple reasons are contributing towards low use of contraceptives including cultural beliefs and myths and fallacies about male contraceptive methods, this is supported by some studies in Pakistan and around the world (Casterline, 2001; IPPF, 2007; Waller et al., 2003; Hardee & Leathy, 2008). Most of those myths and fallacies reported are already known while some were new or at -least not documented in the existing literature. For instance some participants said that use of condoms causes joint pains, this was a new finding.

### 4.1 Condoms

One of the study finding with regards to condom use highlights myths and misconceptions associated with its use. Male youth mostly talked about lack of sexual pleasure associated with the use of condoms which could be a reality too, as documented by some studies (Mosher & Jones, 2010; Rosenberger et al., 2010). However a sexually aware individual should be able to address this issue in a stimulating way and it is up to the service provider and/or promoter to cover these issues during counseling and orientation sessions, and/or in marketing and promotion activities (Jackson, 2004). In a national study in America, no difference was found in sexual arousal and pleasure between condom protected and condom non protected events (Sanders et al., 2010). Some other side effects reported by youths were impotency, dizziness and infections, which appear to be purely myths as condoms usually, do not cause these side effects as evident by scientific studies and research (Waller et al., 2003; Steiner & Cates, 2006). Interestingly, a study conducted by UNFPA on effectiveness of condoms in preventing sexually transmitted infections has also shown similar myths and misperceptions regarding use of condoms and highlights that it is actually a myth that condoms can cause infertility, infections or back pain since no link could be established between the perceived cause and effect (Jackson, 2004). Condom use failure was highlighted by some of the participants, in this regard; education on proper use of condoms can reduce the likelihood of condom failure (Barnett, 1994).

### 4.2 Vasectomy

Both male and female youth had many myths and fallacies about vasectomy. The perception of the participants was that vasectomy causes impairment of physical and mental health, backache, weight gain, physical weakness and impotence. Similar finding have been shown by a study conducted by International Planned Parenthood Federation (IPPF, 2007). In fact men will look and feel the same as before and the operation will not cause them to lose physical strength in any way (IPPF, 2007). In a study conducted in Cebu, Philippines, about 33 percent women thought that their male partners would fail to have erection (Aguilar, 2005). Another one thirds believe that it causes minimize sexual drive while, a quarter of the respondents believed that men lose physical strength after the procedure (Aguilar, 2005). A Harvard Medical School health guide on vaso-vasectomy and vasectomy suggests that the impotence apprehension is baseless as studies have shown that men who experienced impotence after undergoing the procedure are more likely to have female partners who are unable to accept the vasectomy operation (Allen, 2010). The guide also reports that vasectomy has no noticeable impact on a man's ability to perform sexually, or on his sensation of orgasm and pleasure. It does not affect the balance of male hormones, male sex characteristics, or sex drive (Allen, 2010). A study suggests that center of attention should be on social support and counseling of couple for vasectomy to combat the misconceptions regarding it, especially those regarding sexual problems (Mahat, Pacheun & Taechaboonsermsak, 2006). Another study found out that sterilization had less negative impact on physical and psychological performance than the other contraceptive methods (Oddens, 1999). In most cases vasectomy does not have side effect such as impotence, thus vasectomy in families with optimal number of children is recommended (Ahmadipour, 2011).

### 4.3 Recommendations

Our study has explored myths and fallacies about male contraceptive methods, so that findings could be tested in a quantitative study. Main recommendation of this study is to encourage the males to accept condoms and vasectomy use by setting up user's forum for advocacy and removal of myths and fallacies about male contraceptive methods use. Moreover husbands, religious leaders and mothers in law counseling system is required to be introduced to specifically target cultural and gender issues of the youth..

There is a need to disseminate correct information on male contraceptive methods use through easy to understand booklets in local languages for youth of the study area, which could further help in addressing related myths and fallacies about male family planning methods. Moreover, media services could be used to promote the public messages about the benefits of use of these methods.

### 4.4 Strengths and Weaknesses of Study

Strengths of this study are that it addresses the reproductive health issues of youth in a community setting. Moreover focus group discussions were conducted at health houses. Main limitations were that there was difficulty in arranging focus group discussions because of security situation in the city during study period and unmarried youth not included in study, because of cultural constrains. One weakness of the study might be that the data were initially collected in Urdu, translated to English and analyzed in the English version. However, a multidisciplinary team with experienced qualitative researchers took part at all stages of the research process. Integration

## 5. Conclusion

Contraceptives use amongst youth is low. In addition lack of appropriate knowledge about contraceptives contributes to low CPR among the youth. Moreover myths & fallacies about male contraceptive methods were potential factors contributing to low use of contraceptives. Important policy implications are counseling through peers & training of family planning service providers about male contraceptive methods to address myths & fallacies from minds of youth. In addition there is a need for long term planning for educating the women and men.
